# The Complete Mitochondrial Genome of the Foodborne Parasitic Pathogen *Cyclospora cayetanensis*

**DOI:** 10.1371/journal.pone.0128645

**Published:** 2015-06-04

**Authors:** Hediye Nese Cinar, Gopal Gopinath, Karen Jarvis, Helen R. Murphy

**Affiliations:** Food and Drug Administration, Center for Food Safety and Nutrition, Office of Applied Research, Division of Virulence Assessment, Laurel, Maryland, United States of America; University of Florence, ITALY

## Abstract

*Cyclospora cayetanensis* is a human-specific coccidian parasite responsible for several food and water-related outbreaks around the world, including the most recent ones involving over 900 persons in 2013 and 2014 outbreaks in the USA. Multicopy organellar DNA such as mitochondrion genomes have been particularly informative for detection and genetic traceback analysis in other parasites. We sequenced the *C*. *cayetanensis* genomic DNA obtained from stool samples from patients infected with *Cyclospora* in Nepal using the Illumina MiSeq platform. By bioinformatically filtering out the metagenomic reads of non-coccidian origin sequences and concentrating the reads by targeted alignment, we were able to obtain contigs containing *Eimeria*-like mitochondrial, apicoplastic and some chromosomal genomic fragments. A mitochondrial genomic sequence was assembled and confirmed by cloning and sequencing targeted PCR products amplified from *Cyclospora* DNA using primers based on our draft assembly sequence. The results show that the *C*. *cayetanensis* mitochondrion genome is 6274 bp in length, with 33% GC content, and likely exists in concatemeric arrays as in *Eimeria* mitochondrial genomes. Phylogenetic analysis of the *C*. *cayetanensis* mitochondrial genome places this organism in a tight cluster with *Eimeria* species. The mitochondrial genome of *C*. *cayetanensis* contains three protein coding genes, cytochrome (*cytb*), cytochrome *C* oxidase subunit 1 (*cox1*), and cytochrome *C* oxidase subunit 3 (*cox3*), in addition to 14 large subunit (LSU) and nine small subunit (SSU) fragmented rRNA genes.

## Introduction

*C*. *cayetanensis* is an emerging human pathogen causing gastrointestinal disease in humans around the world and is acquired through food and waterborne transmission, often by consumption of contaminated fresh produce [1–4]. In the United States, *Cyclospora* was the etiological agent of recent food-borne outbreaks affecting more than nine hundred people in 2013 and 2014 (http://www.cdc.gov/parasites/cyclosporiasis/outbreaks/index.html). *Cyclospora* belongs to the phylum Apicomplexa, which is a large group of protists, related to dinoflagellates and ciliates [5]. Other members of Apicomplexa include *Plasmodium*, *Babesia*, *Theileria*, *Toxoplasma*, *Eimeria*, and *Cryptosporidium*.

Mitochondria are organelles essential for cellular processes such as energy metabolism, signaling, cellular differentiation and cell death [6]. Mitochondria contain their own genomes referred to as mitochondrial (mt) genomes [7,8]. Most apicomplexan species possess mt genomes of ~6kb in size with considerable variability in structure and organization [9]. The genomes of apicomplexan mitochondria encode three mt protein coding genes (cytochrome *c* oxidase subunits, *cox1* and *cox3*, and cytochrome *b*, *cob*), in addition to highly fragmented rRNA genes. These mt genomes exist as either monomeric or concatemeric linear forms [10]. Among the apicomplexan organisms, *Eimeria* is the phylogenetically closest genus to *Cyclospora* based on 18S gene sequences [11] [12]. The complete mitochondrial genome sequences for 13 *Eimeria* species have been reported [13–15], all of which range from 6.1 to 6.4 kb in size with highly conserved content and structure [13].

Despite the significant clinical and public health importance of *C*. *cayetanensis*, very little sequence information is available for this organism. Lack of a laboratory culture method for *Cyclospora* [16] has hampered the ability to obtain large numbers of highly purified oocysts and sufficient *Cyclospora* DNA for genomics studies. Nevertheless, because of recent advances in next generation sequencing technologies, we performed whole genome sequencing (WGS) of *C*. *cayetanenesis* using genomic DNA samples extracted from oocysts purified from human fecal samples. The sequence output was metagenomic in nature, but we were able to obtain contigs containing *Eimeria*-like mitochondrial, apicoplastic and some chromosomal genomic fragments by bioinformatically filtering out the reads of non-coccidian origin sequences.

Here we report the sequence, gene organization, and structure of the *C*. *cayetanensis* mt genome from *Cyclospora* originating in Nepal. Our data show that the *C*. *cayetanensis* mitochondrial genome is organized as a concatemeric linear 6.3 kb molecule which is closely related to the mt genomes of *Eimeria* species.

## Materials and Methods

### Cyclospora

Human stool samples containing *C*. *cayetanensis* oocysts were obtained from the Microbiology and Public Health Research Laboratory at Tribhuvan University Teaching Hospital in Kathmandu, Nepal and the University of Georgia in Athens, Georgia, USA (This study was reviewed and approved by Institutional review Board of FDA, RIHSC- ID#10-095F). *Cyclospora* oocysts were purified by a method similar to the one used for *Cryptosporidium* [17](Arrowood and Donaldson 1996). Briefly, *Cyclospora* oocysts were recovered from sieved fecal samples by differential sucrose and cesium chloride gradient centrifugations. *Cyclospora* oocysts were counted using a haemocytometer and a Zeiss Axio Imager D1 microscope with an HBO mercury short arc lamp and a UV filter (350 nm excitation and 450 nm emission).

### Sequencing

Genomic DNA was isolated from purified *C*. *cayetanensis* oocyst preparations using the ZR Fecal DNA MiniPrep kit (Zymo Research, Irvine, CA) following the manufacturer’s instructions. DNA concentration was measured with a Qubit 1.0 Fluorimeter using the Qubit dsDNA HS Assay Kit (Life Technologies, Grand Island, NY). Shotgun sequencing of genomic DNA isolated from three oocyst preparations purified from two different fecal samples was performed on the Illumina MiSeq platform (Illumina, San Diego, CA) using the Nextera XT library preparation kit (Illumina, San Diego, CA). Approximately 12 pmol of each library was paired-end sequenced (2X 250 cycles) on the MiSeq.

### Bioinformatic analysis

Metagenomic profiling of the shotgun datasets was carried out by MetaPhlAn (PMID: 2688413) using default parameters [18]. A local database of apicomplexan and dinoflagellates genomes from *Cryptosporidium*, *Perkinsus* and *Eimeria* was created using sequences downloaded from NCBI (PMID: 25398906) and used for alignment with Bowtie2 [19]. This strategy filtered sequence reads specific to Apicomplexa from the metagenomic runs by collecting those sequences that mapped to the target apicomplexan genome database. CLCworkbench 6 (http://www.clcbio.com/products/clc-genomics-workbench/) was used to generate *de novo* assembly of sequences to obtain a final set of contigs of various lengths, and to map the filtered reads back to the apicomplexan genomic regions.

### Confirmation of the mitochondrial genome structure via PCR amplification and re-sequencing

PCR reactions were performed using the Platinum PCR SuperMix High Fidelity kit (Invitrogen, Grand Island, NY, USA) according to the manufacturer's instructions. Genomic DNA extracted from *C*. *cayetanensis* oocysts was used (100 pg) as the template. The PCR primers (Table 1) were designed to cover the entire sequence of the longest mitochondrial contig (6.3 kb) generated. The PCR products were gel purified using the QIAGEN Gel Extraction kit, and sequenced either directly or after cloning using the TOPO TA cloning kit with One Shot TOP10 Chemically Competent *E*.*coli* (Invitrogen, USA). DNA sequences were trimmed and assembled using the CodonCode Aligner version 5.0 (CodonCode Corporation, Centerville, MA). WebACT tool [20], a web version of Artemis genome comparison tool [21] was used to visualize the mappings of PCR products to the genome assemblies generated by NCBI BLAST.

**Table 1:** Primer sequences used for PCR.

Primers	Sequence (5’-3’)
**1F**	GGTACTACATCAGCTTCTATGG
**1R**	GCGTGGAAAGGTTCCCGTGAG
**3Fb**	GTTGGAGCTCAATTACCTCAAGAAG
**3Rb**	TTTGTATGGATTTCACGGTCAACTC
**4Fb**	CCATTTCGTACTATCTCTTGGTGCG
**4Rb**	CTAATACAGTGAGCAAGAATGGTGA
**4B-F**	GCTGGAAGACGGAATCGTTAC
**4B-R**	CAAATAGTAGTAACTCAGTAC
**2F-begin**	GACAAAGTCCCAGCAGTAGC
**2R**	CTTTATGGTTTGCAGCAGTTAG
**5F**	CAGGAGCTCTGATCCTTGAATTCTAT
**5R**	GCACAAGCACTCAGCAAGTTAAGAGAATG
**End6R**	CACATGATGCTCCAGTAGCATGTAGG
**2Fb**	CATTCATGCAGGACGGAATTTACC
**End5R**	GCAATCGGATCGTGTTGGCTAGGTGTAC
**End1F**	GGCGTGTAGAGCATCTTTATCTAGGTAG
**End2F**	CTGTCCGAAACAATAACCTTAGGC
**End3F**	CTACCAAAGCATCCATCTACAGCTG
**End4F**	GCATCCATCTACAGCTGCGGAAACTG

### Mitochondrial sequence analysis

The mt genome sequences for thirteen *Eimeria* species and *P*. *falciparum* as an outlier were downloaded from NCBI and used for comparative analysis. The accession numbers of the sequences used are as follows: *E*. *acervulina-*HQ702479; *E*. *meleagridis-*KJ608418; *E*. *meleagrimitis-*KJ608414; *E*. *dispersa-*KJ608416; *E*. *adenoeides-*KJ608415; *E*. *gallopavonis-*KJ608413; *E*. *magna-*KF419217; *E*. *mitis-*JN864949; *E*. *tenella-*HQ702484; *E*. *praecox-*HQ702483; *E*. *necatrix-*HQ702482; *E*. *maxima-*HQ702481; *E*. *brunetti-* HQ702480; and *P*. *falciparaum NF54-*AJ27684.

The 5’ and 3’ ends of the *C*. *cayetanensis* mitochondrial sequences were manually curated by comparing the initial assembly with complete mitochondrial genomes from *Eimeria* as reference to obtain the 6,274 bp long complete sequence. The *Eimeria and Cyclospora* mt sequences were aligned with MEGA6 suite [22] using ClustalW [23]. A phylogenetic tree was built using using default parameters in Maximum Likelihood algorithm options for the 14 genomes. Mitochondrial genome from *P*.*falciparum* was initially used as an outgroup. Test of phylogeny was conducted on Mega 6 using 500 replicates for bootstrap analysis. The 6274 bp long mitochondrial genome from *C*. *cayatenensis* was annotated using Genbank record KJ608417 submitted to NCBI and assigned accession number KP231180.

## Results and Discussion

### Assembly of *C*. *cayetanensis* mitochondrial genome sequence

Metaphlan analysis of the quality-filtered data revealed that more than 98% of our reads mapped to fecal bacterial sequences. To identify the apicomplexan sequences, we first designed a custom database with genome sequences from *Eimeria*, *Perkinsus* and other related apicomplexans, and mapped our sequence reads against this database identifying 47,000 apicomplexan-specific reads that assemble into 482 contigs. From these, we identified a 6.3 kb long sequence which is highly similar to those mt sequences from *Eimeria* (average BLASTn score 91–93%).

### The complete sequence of *C*. *cayatenensis* mitochondrial genome

**Fig 1:**
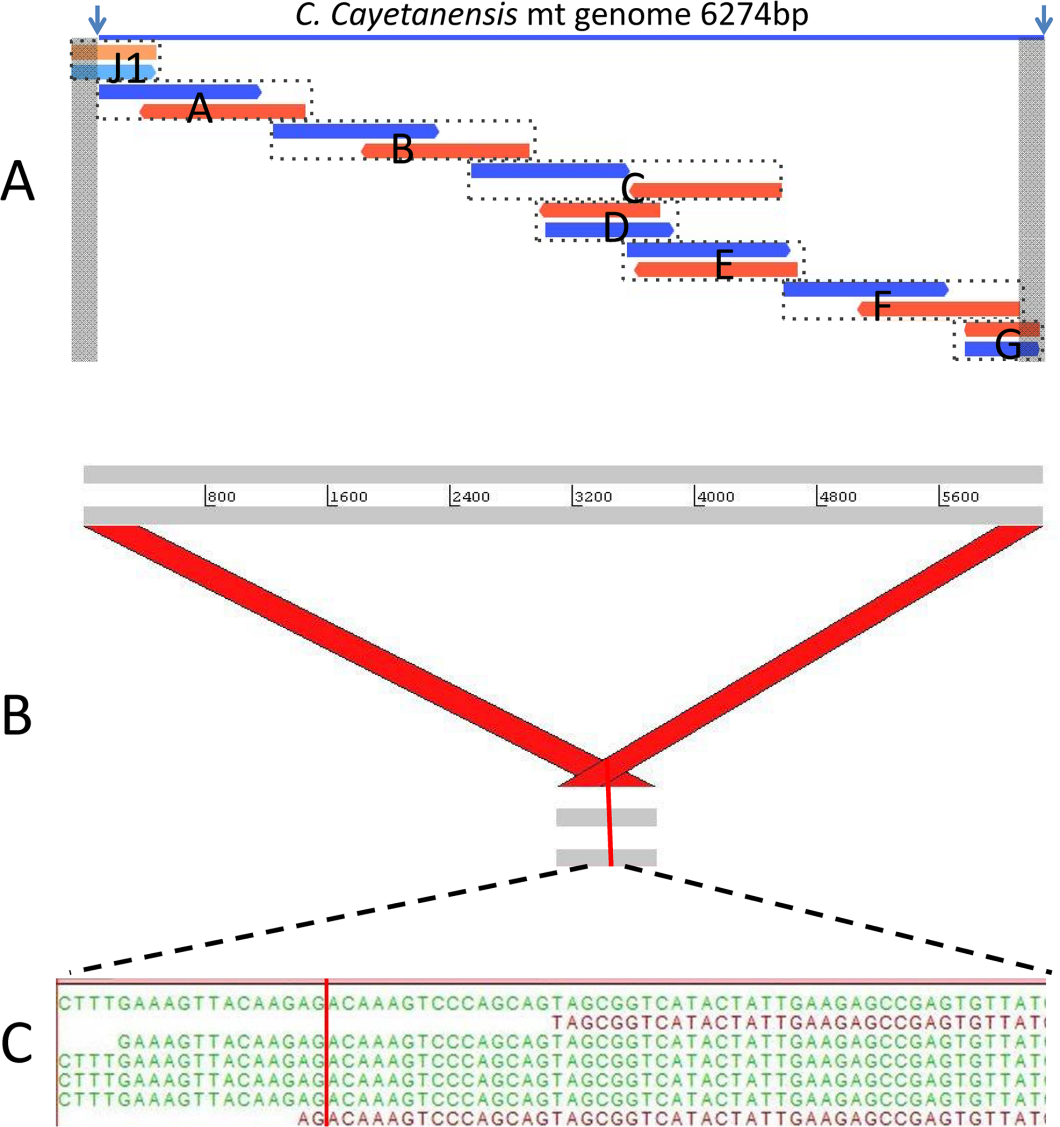
Confirmation of the *C*. *Cayetanensis* mt genome sequence. A) Overlapping PCR fragments used to confirm the *C*. *cayetanenesis* mt genome sequence and concatemeric structure. PCR fragments are shown with dotted lines. Forward sequence reads are shown in blue or orange and reverse reads are shown in red or light blue. Terminal grey bands indicate identical sequence regions. B) A 659 bp contig constructed using overlapping junction PCR fragment sequences (see Table 2) is represented in gray. Diagonal red bars represent portions of the junction PCR product mapping to the ends of the complete *C*. *cayetanensis* mt genome. C) WGS reads spanning the junction region in the initial mt assembly. The vertical red lines mark the tail:head junction.

By aligning the initial *Cyclospora* 6.3 kb contig assembly with the *E*. *tenella* mt genome, we were able to delineate the terminal bases of the molecule *in silico*. Next, we sequenced the PCR products amplified from *Cyclospora* oocysts using seven primer sets covering the whole 6.3 kb-long assembled sequence (Fig 1 and Table 2), confirming and closing the complete *C*. *cayatenensis* mt genome (Acc: KP231180). When compared with *Eimeria* mt sequences using ClustalW, the *Cyclospora* mt genome aligned well with 13 available Eimerid mitochondrial genomes in sequence and organization. In particular, a front block of about 200 bases and a large stretch of sequences towards the end are highly conserved in *Eimeria* and *Cyclospora* genomes, suggesting a common ancestral mt genome (S1 File).

**Table 2:** PCR primer sets and amplicons.

**Full length mt genome PCR**			
**Amplicons**	**Forward Primer**	**Reverse Primer**	**Amplicon size (bp)**
**A**	2F-Begin	2R	1393
**B**	3Fb	3Rb	1609
**C**	4Fb	4Rb	1984
**D**	5F	5R	901
**E**	4B-F	4B-R	1022
**F**	1F	1R	1507
**G**	2Fb	End5-R	458
**Junction PCR**			
**J1**	End1-F	End6-R	634
**J2**	2Fb	End5-R	458
**J3**	End2-F	End6-R	603
**J4**	End3-F	End6-R	541
**J5**	End4-F	End6-R	535

In the phylum Apicomplexa, mitochondrial genomes have either concatemeric or monomeric linear structures as represented by *Eimeria*, and *Piroplasms*, respectively [10]. To determine the *C*. *cayetanensis* mt genome structure, we amplified, sequenced and assembled the PCR products designed to span the tail to head junction (Table 2, Fig 1A). The resulting 659 bp sequence mapped to the terminal ends of the *C*. *cayatenensis* mt genome as expected for a structure with tail to head junction (Fig 1B). We were able to identify a few apicomplexan-specific metagenomic reads that spanned across this junction region as shown in the Fig 1C. Our results are consistent with a concatemeric structure that is the hallmark of the closely related *Eimeria* mt genomes; however, we cannot rule out a circular mt genome structure.

**Fig 2:**
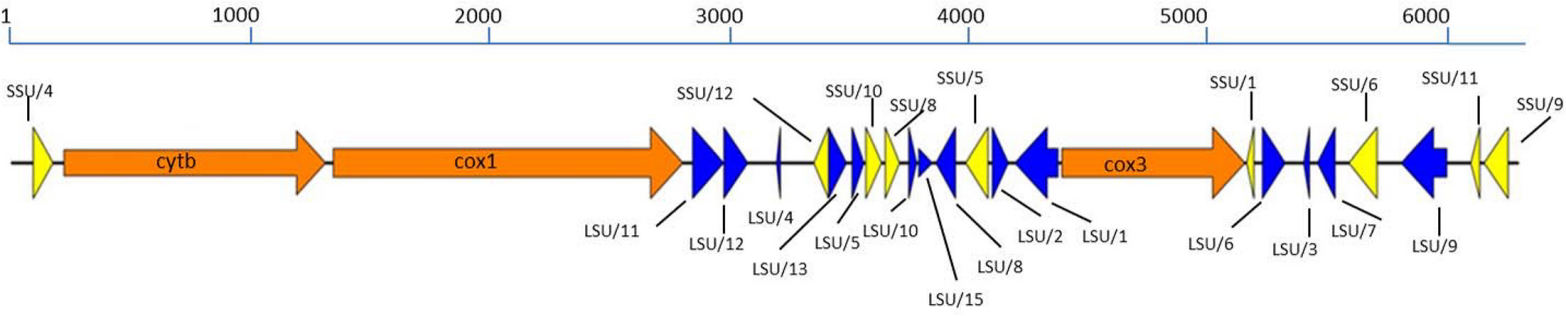
Gene arrangement of the *C*. *cayetanensis* mt genome. Predicted genes on the *Cyclospora* mitochondrial genomes were derived based on ClustalW alignment with the Eimeria gallopavonis (KJ608417). Orange indicates coding genes, blue indicates fragments of LSU rRNA, and yellow indicates fragments of SSU rRNA. Transcriptional direction is indicated by arrowed end.

The complete *C*. *cayetanensis* mt genome is 6,274 bp, with a base composition of A (30%), T (36%), C (16%), and G (17%). Based on the available annotations in the NCBI for *Eimeria gallopavonis*, *Cyclospora* mitochondrian genome was annotated after aligning the two genomes. The *C*. *cayetanensis* mt genome encodes three protein-coding genes; *cytb*, 1080 bp [128 bp-1207 bp], ATG start codon; *cox1*, 1443 bp [1248 bp- 2690 bp], GTT start codon; and *cox3*, 780 bp [4226 bp- 5005 bp], ATT start codon. In addition to these three protein-coding genes, twelve large-subunit (LSU), and seven small-subunit (SSU) fragmented rRNA genes are present in the mt genome (Fig 2). Pairwise amino acid sequence alignments between the individual protein coding genes of *C*. *cayetanensis* mt genome, and the corresponding coding genes of seventeen other published *Eimeria* mt genomes revealed sequence identities that ranged from 90–97% for *cytbB*, 93–97% for *cox1*, and 83–93% for *cox3*.

### Phylogeny

**Fig 3:**
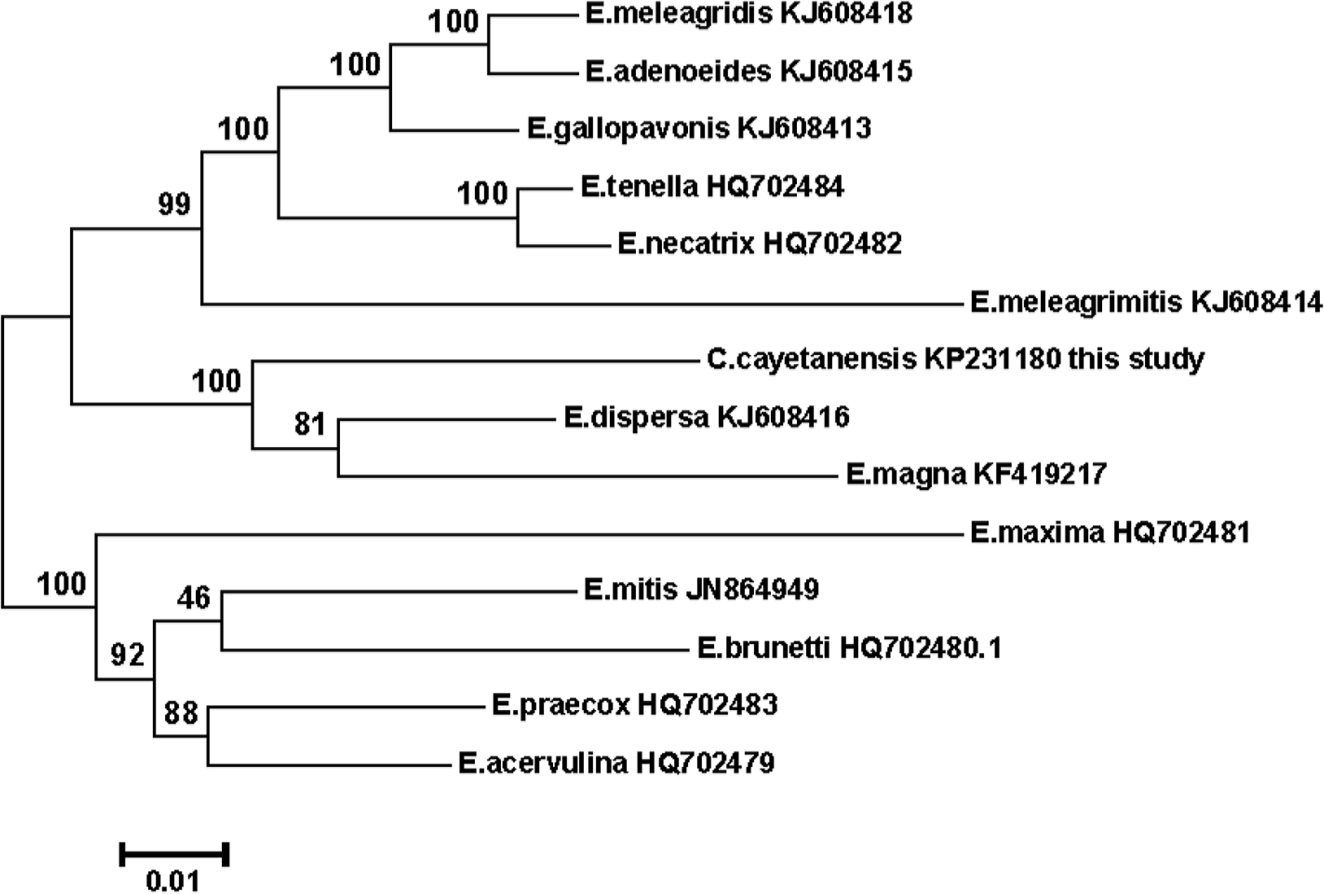
Phylogenetic relationships among *Eimeria* species and *C*. *cayetanensis* based on mitochondrial genome sequences. NCBI accession numbers are included. The evolutionary relationship between the *Eimeria* (13), *Cyclospora(1)* and *P*.*falciparum* (1) mitochondrial genomes was inferred using the Maximum Likelihood method with 500 replications for bootstrap analysis. The confidence value for clustering is given as percentage above the branches. The scale bar points to the number of substitutions per site. After analysis, the outgroup branch was removed for clarity. Default parameters in MEGA6 were retained for phylogenetic analysis and tree building.

Mt genomes from thirteen species of *Eimeria* and from a strain NF54 of *P*. *falciparum* were aligned with the *C*. *cayetenensis* mt genome for Maximum Likelihood (ML) phylogenetic reconstruction analysis (Fig 3). The mt genome nucleotide phylogeny of *C*. *cayetenensis* supports a closer relationship to *Eimeria* species *E*. *magna* and *E*. *dispersa* that infect rabbits and turkeys, respectively. *C*. *cayetenensis* appears to infect only humans [16] and, notably, we found that the *C*. *cayetenensis* mt sequences fall into a clade that contains the rabbit infecting *E*. *magna* (Fig 3). Avian infecting *Eimeria* species in our comparison clustered into two other distinct clades (Fig 3). The cladistic distribution of the two groups of *Eimeria* spp. followed mostly the pattern described in Barta 2014 [15]. The ‘turkey *Eimerids’* that infect the caeca of turkeys *(E*. *meleagridis*, *E*. *meleagrimitis*, *E*. *adenoeides*, and *E*. *gallopavonis)* formed a clade that included *E*. *tenella* and *E*. *necatrix (*chicken *Eimerids* that infect the caeca) as sister groups. The other ‘chicken *Eimerids’ (E*. *mitis*, *E*. *praecox*, *E*.*acervulina*, *E*.*maxima* and *E*. *brunette)* grouped together. *C*. *cayatenensis* clustered together with rabbit *Eimerid*, *E*.*magna*, and a turkey infecting strain, *E*. *dispersa*. Previous studies using 18S rRNA [24–26]and ITS-1 sequences [27]place *C*. *cayetanensis* into a clade with chicken eimerids. We constructed a phylogenetic tree using currently available *Eimeria* spp.18S rRNA gene sequences, and *C*. *cayetanensis* 18S rRNA gene (S1 Fig). In our 18S rRNA bootstrap analysis, *C*. *cayetanensis* appears to cluster with *E*. *meleagrimitis*. Mammalian coccidians *E*. *magna* and *E*. *bovis* are found to be closely related to each other, but located in a clade different from *C*. *cayetanensis* tree location. The monophyletic grouping of mammalian *Coccidians (Cyclospora* and *E*. *magna)* in mitochondrial phylogenetic tree warrants further analysis, though beyond the scope of this work, preferably using apicomplexan apicoplastic and chromosomal genome sequences.

## Conclusions

Our data suggest that the *C*. *cayetanensis* mt genome is a 6.3 kb linear molecule with a concatemeric structure. The content and order of protein-coding, and rRNA genes are highly conserved with those found in *Eimera* spp. The *Cyclospora* mt genome shows a close phylogenetic relationship with that of mammalian-infecting *E*. *magna*. Our study potentially opens the way for studies aimed toward development of organellar genome single nucleotide polymorphism (SNP) based trace-back assays for investigation of *Cyclospora* outbreaks, an approach successfully employed for the epidemiology of *P*. *falciparum* [28–30].

## Supporting Information

S1 Fig Phylogenetic relationships among *Eimeria* species and *C*. *cayetanensis* based on 18S rRNA gene sequences. 18S rRNA sequence files from NCBI were used to infer evolutionary relationship between 22 *Eimeria* spp. and *C*. *cayatenensis* strain. The bootstrap analysis was carried out using the Maximum Likelihood method with 500 replications. The confidence value for the resultant clusters is given as percentage above the branches. The scale bar points to the number of substitutions per site. The analysis and the tree building were carried out using default parameters in MEGA 6 software.
(TIF)

S1 File Sequences of 13 *Eimeria* mitochondrial genomes were aligned with the *Cyclospora* mitochondrial sequence from this study using ClustalW tool in the MEGA6 suite. 87 bp in some of the Eimeria sequences were cut and appended to the 3’ end of the respective sequence files. ClustalW identified blocks of highly conserved 5’ and 3’ ends of *Eimeria* and *Cyclospora* mitochondrial sequences.
(TXT)
