# Expression of Concern: The Complete Mitochondrial Genome of the Foodborne Parasitic Pathogen *Cyclospora cayetanensis*

**DOI:** 10.1371/journal.pone.0282228

**Published:** 2023-02-21

**Authors:** The PLOS ONE Editors

After this article [1] was published, concerns were raised about the approval for use of the biological samples obtained from Nepal.

This study involved human stool samples that originated in Nepal. The samples were collected at Tribhuvan University Teaching Hospital, Microbiology and Public Health Research Laboratory, Kathmandu, Nepal; transferred to the University of Georgia in 2012; and provided to the US Food and Drug Administration (FDA) in 2015. As of the time of this notice, PLOS has been unable to reach the National Health Research Council (NHRC) in Nepal to clarify if the study complied with Nepalese regulations for human subjects research and/or transfer of human fecal samples from Nepal to the University of Georgia and then to the FDA.

A protocol for collection and analysis of anonymized fecal specimens for the isolation of *Cyclospora cayetanensis* oocysts was approved by the FDA Research Involving Human Subjects Committee (RIHSC) in 2010–2013 (#10-095F). However, neither University of Georgia nor Tribhuvan Teaching Hospital were specified as potential sources for specimens in the RIHSC approval document; instead, another US-based institution was named. This discrepancy has not yet been resolved. PLOS has contacted the FDA.

The University of Georgia Office of Research evaluated the study’s compliance with applicable policies, regulations, and guidance, and noted that University of Georgia IRB concluded the study neither qualified as human subjects research (as defined in the Code of Federal Regulations 45 CFR 46.102) nor required further IRB review or approval. The project received approval of the University of Georgia Institutional Biosafety Committee in 2010 and 2015. The University of Georgia IRB and Biosafety Committee reviewed research conducted with these samples at the University of Georgia. The work conducted at the FDA and the use of the samples at the FDA were not assessed by the University of Georgia committees.

The *PLOS ONE* Editors issue this Expression of Concern to notify readers of the unresolved issues discussed above.
